# Evidence accumulation is biased by motivation: A computational account

**DOI:** 10.1371/journal.pcbi.1007089

**Published:** 2019-06-27

**Authors:** Filip Gesiarz, Donal Cahill, Tali Sharot

**Affiliations:** 1 Affective Brain Lab, Department of Experimental Psychology, University College London, London, United Kingdom; 2 Google, Mountain View, California, United States of America; McGill, CANADA

## Abstract

To make good judgments people gather information. An important problem an agent needs to solve is when to continue sampling data and when to stop gathering evidence. We examine whether and how the desire to hold a certain belief influences the amount of information participants require to form that belief. Participants completed a sequential sampling task in which they were incentivized to accurately judge whether they were in a desirable state, which was associated with greater rewards than losses, or an undesirable state, which was associated with greater losses than rewards. While one state was better than the other, participants had no control over which they were in, and to maximize rewards they had to maximize accuracy. Results show that participants’ judgments were biased towards believing they were in the desirable state. They required a smaller proportion of supporting evidence to reach that conclusion and ceased gathering samples earlier when reaching the desirable conclusion. The findings were replicated in an additional sample of participants. To examine how this behavior was generated we modeled the data using a drift-diffusion model. This enabled us to assess two potential mechanisms which could be underlying the behavior: (i) a valence-dependent response bias and/or (ii) a valence-dependent process bias. We found that a valence-dependent model, with both a response bias and a process bias, fit the data better than a range of other alternatives, including valence-independent models and models with only a response or process bias. Moreover, the valence-dependent model provided better out-of-sample prediction accuracy than the valence-independent model. Our results provide an account for how the motivation to hold a certain belief decreases the need for supporting evidence. The findings also highlight the advantage of incorporating valence into evidence accumulation models to better explain and predict behavior.

## Author summary

People tend to gather information before making judgments. As information is often unlimited a decision has to be made as to when the data is sufficient to reach a conclusion. Here, we show that the decision to stop gathering data is influenced by whether the data points towards the desired conclusion. Importantly, we characterize the factors that generate this behaviour using a valence-dependent evidence accumulation model. In a sequential sampling task participants sampled less evidence before reaching a desirable than undesirable conclusion. Despite being incentivized for accuracy, participants’judgments were biased towards believing they were in a desirable state. Fitting the data to an evidence accumulation model revealed this behavior was due both to the starting point and rate of evidence accumulation being biased towards desirable beliefs. Our results show that evidence accumulation is altered by what people want to believe and provide an account for how this modulation is generated.

## Introduction

Judgments are formed over time as information is accumulated [1–3]. When given an opportunity to sample unlimited data an individual can decide to continue gathering evidence until a certain threshold is reached [4,5]. This decision involves the trade-off between time and accuracy–an exchange that has been well-studied [6–8].

It seems probable, however, that the decision to stop gathering evidence would also be influenced by the desire to hold one belief over another [9, 10]. For example, people are less likely to seek a second medical opinion when the first physician delivers good news than when she delivered bad news [11]. The problem with such observations is that they often confound desirability with probability–a patient might seek a second opinion after receiving a dire diagnosis simply because the diagnosis is rare (and thus seems unlikely), not because it is undesirable.

Here, we set out to empirically examine in a controlled laboratory setting whether and how the desire to hold a belief influences the amount of information required to reach it, when all else is held equal. Presently, we have limited understanding if and how motivation alters evidence accumulation, despite the potential for such effects to dramatically impact people’s decisions in domains ranging from finance to politics and health [9–11]. To gain insight into the underlying process we tease apart the computational elements that may be influenced by motivation.

Specifically, we hypothesized that the desire to hold one judgment over another could alter information accumulation in at least two ways. First, people may be predisposed towards desired judgments before observing any evidence at all (for example, one may believe it will be a nice day before checking the weather or glancing outside) [12]. A second, not mutually exclusive possibility is that a desirable piece of evidence (e.g., a ray of sunlight) drives beliefs towards a desirable judgment (‘it will be a nice day’), more so than an undesirable piece of evidence (e.g., the sound of rain) towards an undesirable judgment (‘it will be a grey day’) [13]. These two distinct mechanisms will result in the same observable behavior. In particular, less information will be gathered to support desirable judgments than undesirable, such that the former would be reached faster.

To dissociate these mechanisms, we use a computational approach. We adopt a sequential sampling model to model noisy evidence accumulation towards either of two decision thresholds [1,14,15]. The model allows estimating both (i) the starting point and (ii) rate of evidence accumulation, reflecting the quality of information processing [14]. This enables us to ask if either of these factors, or both, are influenced by motivation.

In our task participants witness various events that are contingent upon which one of two hidden states they are in. One state was associated with greater rewards than losses (desirable state) and the other with greater losses than rewards (undesirable state). The participants had no control over which state they were in; their task was simply to judge the state, gaining additional rewards for accurate judgments and losing rewards for inaccurate judgments. Thus, it is in participants’ best interest to be as accurate as possible and they were allowed to accumulate as much evidence as they wish before making a judgment. We examine whether and how the accumulation process is sensitive to participants’ motivation to believe that they are in one state and not the other.

## Results

**Fig 1:**
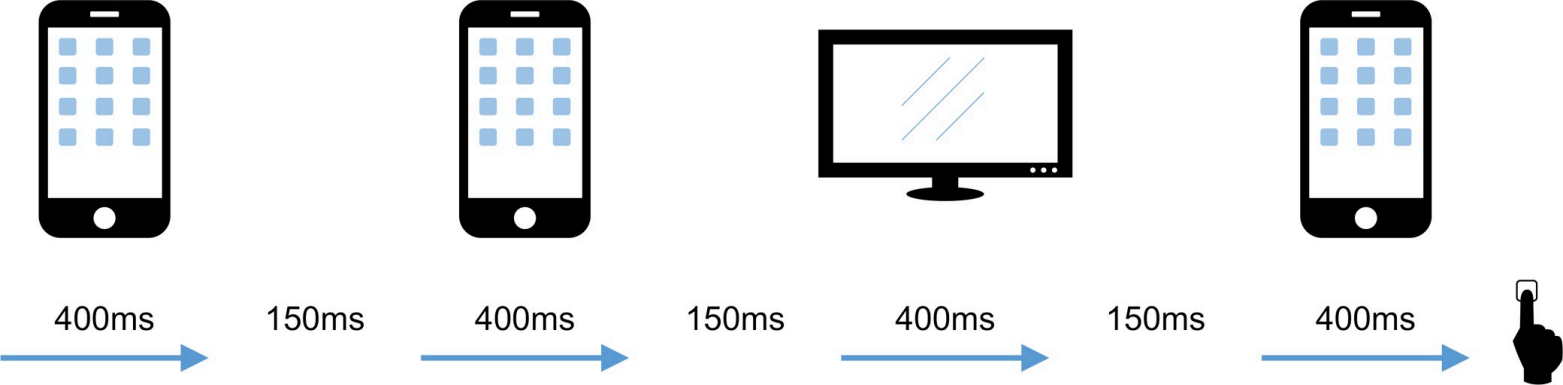
Task. On each trial participants saw TVs and phones moving along the screen and had to guess if they were in a TV factory (that sometimes produces telephones) or a phone factory (that sometimes produces TVs). They were incentivized for accuracy and could enter their judgment whenever they liked. Each participant was “invested” in one factory. On trials where they happened to be in that (desirable) factory they gained points, on trials in which they happened to be in the other (undesirable) factory they lost points.

We tested 84 participants on “The Factory Game” (**Fig 1**). For each trial in this game, participants saw a series of telephones and televisions that ran across a conveyor belt on screen. Their task was to decide whether the series was being generated by a telephone factory (which mostly produced telephones, but sometimes produced televisions) or a television factory (which mostly produced televisions, but sometimes produced telephones). They received a reward for being accurate and a penalty for being inaccurate. The reward and penalty amounts were unspecified and said to differ on each trial, preventing participants from using any strategies based on a computation of an exact expected value.

Additionally, participants were told that they had “invested” in either a telephone or television factory. In the context of the game, this meant that they received a bonus payment when they happened to be visiting the type of factory they had invested in (*desirable* factory trials) and a penalty when visiting a factory they had not invested in (*undesirable* factory trials). The amount of points received or lost for being in a desirable and undesirable factory was not specified and said to differ on each trial. Crucially, this bonus/loss *was not dependent upon their judgment*, so even though it was preferable to be visiting a rewarding factory, there was no incentive to bias their judgment in that way. We ensured that participant understood this by implementing comprehension questions.

We also ran a replication and extension study (N = 92), which is described in **Supplementary Information**. The results of this second study replicate the behavioral and modeling results described below.

### Participants are more likely to conclude they are in a desirable factory than undesirable factory and require weaker evidence to do so

**Fig 2:**
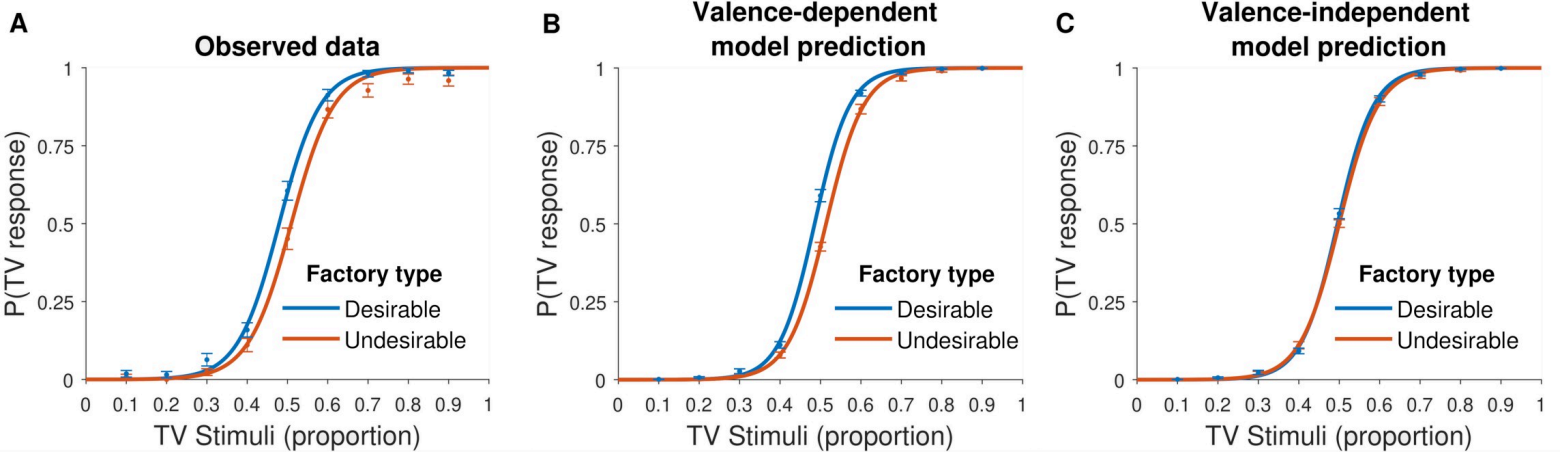
Participants require weaker supporting evidence to reach a desirable conclusion, a pattern that is reproduced by the valence-dependent model. Fitted psychometric function on (A) participants’ data reveals that the probability of judging a factory as a TV factory increases with proportion of TVs observed. Importantly, a smaller proportion of TVs is needed to judge a factory as a TV factory when the factory is desirable than when it is undesirable. (B) The same pattern is observed when plotting simulated data generated from winning model 4 (see Table 1) in which both the starting point of the accumulation and the drift rate are valence-dependent, but not when (c) plotting simulated data generated from a valence-independent model, where starting point and drift rate are not modulated by valence.

The proportion of factories participants judged as desirable was significantly greater than the number they actually encountered (mean = 53.7%, t(83) = 3.42, p < 0.0001). They gathered less samples before concluding they were in a desirable than undesirable factory (t(83) = -3.10, p < 0.01) and required a smaller proportion of samples to be consistent with their judgment when reaching that conclusion. The latter point is shown by fitting a psychometric function to the data which relates the percentage of TVs observed on a trial to participants’ judgment on whether they are visiting a TV or telephone factory. This was done separately for participants for whom the TV factory was desirable and for whom it was undesirable. As expected, both functions show that the greater the proportion of TVs on a trial the more likely participants are to judge the factory as a TV factory (TV factory desirable: β_1_ = 25.24, 95% CI [21.20, 29.28], TV factory undesirable: β_1_ = 24.34, 95% CI [20.81, 27.88]). Crucially, as can be observed in **Fig 2A,** the psychometric function of participants for whom the TV factory was desirable (blue line) was shifted left compared to the psychometric function of participants for whom the TV factory was undesirable (red line). This means that for the same proportion of TV stimuli participants are more likely to judge they are in the TV factory if the TV factory is desirable than undesirable (indifference parameter was higher when the TV factory was desirable: β_0_ = 0.28, 95% CI [0.05, 0.50] than undesirable: β_0_ = -0.35, 95% CI [-0.60, -0.23]).

As participants concluded they were in a desirable factory more often than undesirable factory, they were more likely to falsely believe they were in a desirable factory when in an undesirable factory (30.96% of undesirable factories wrongly categorized) than to falsely believe they were in an undesirable factory when in a desirable factory (only 24.78% of desirable factories wrongly categorized), *t*(83) = 4.85, *p* < 0.0001. Put another way, a larger proportion of desirable factories were correctly categorized than undesirable factories. Note, however, that desirable and undesirable *responses* did not differ in accuracy (t(83) = -0.63, p = 0.53), nor were these responses different in their speed-accuracy trade-off. In particular, we divided trials to fast and slow for each participant based on their median reaction time. We then calculated the proportion of accurate fast responses and accurate slow responses separately when participants concluded they were in a desirable and undesirable factory. These proportions were then subjected to a 2 (speed: fast/slow) by 2 (response: desirable/undesirable) ANOVA. We found a main effect of speed on accuracy, with slow responses being more accurate than fast responses (F = 24.88, p < 0.0001). However, as mentioned above there was no effect of response desirability on accuracy (F = 0.46, p = 0.50), nor an interaction between response desirability and speed (F = 1.13, p = 0.29).

In sum, the results show that participants were more likely to believe they were in a desirable factory. They gathered less samples before making these judgments and required a smaller proportion of the samples to be consistent with said belief. We next sought to understand how this behavior was generated by characterizing the underlying computations that give rise to the behavior. In particular, the bias we observed may have emerged if valence was modulating (i) the starting point of the accumulation process; (ii) the rate of evidence accumulation; or (iii) both. To tease apart these possible mechanisms we modeled the data as a drift-diffusion process.

#### Starting point and drift rate are valence-dependent

Responses were modeled as a drift-diffusion process [1, 14, 15] with the following parameters: (1) *t0—*amount of non-accumulation time; (2) *a*—distance between decision thresholds; (3) *z—*starting point of the accumulation process; and (4) *v*–drift rate. The drift rate is the rate of evidence accumulation, which we allowed to vary on a trial-by-trial basis depending on the consistency of evidence (see Methods). We ran six models in total. In models 1,2,5 the starting point was fixed to 0.5, while in models 3,4,6 we allowed the starting point to vary (thus allowing a starting point bias). In models 2,4,5,6, we allowed the drift rate to vary depending upon whether the participant was visiting a desirable factory or an undesirable factory (thus allowing a process bias). In addition, models 5 and 6 allowed the process bias to interact with the difficulty of the trial. See Method for further details.

**Fig 3:**
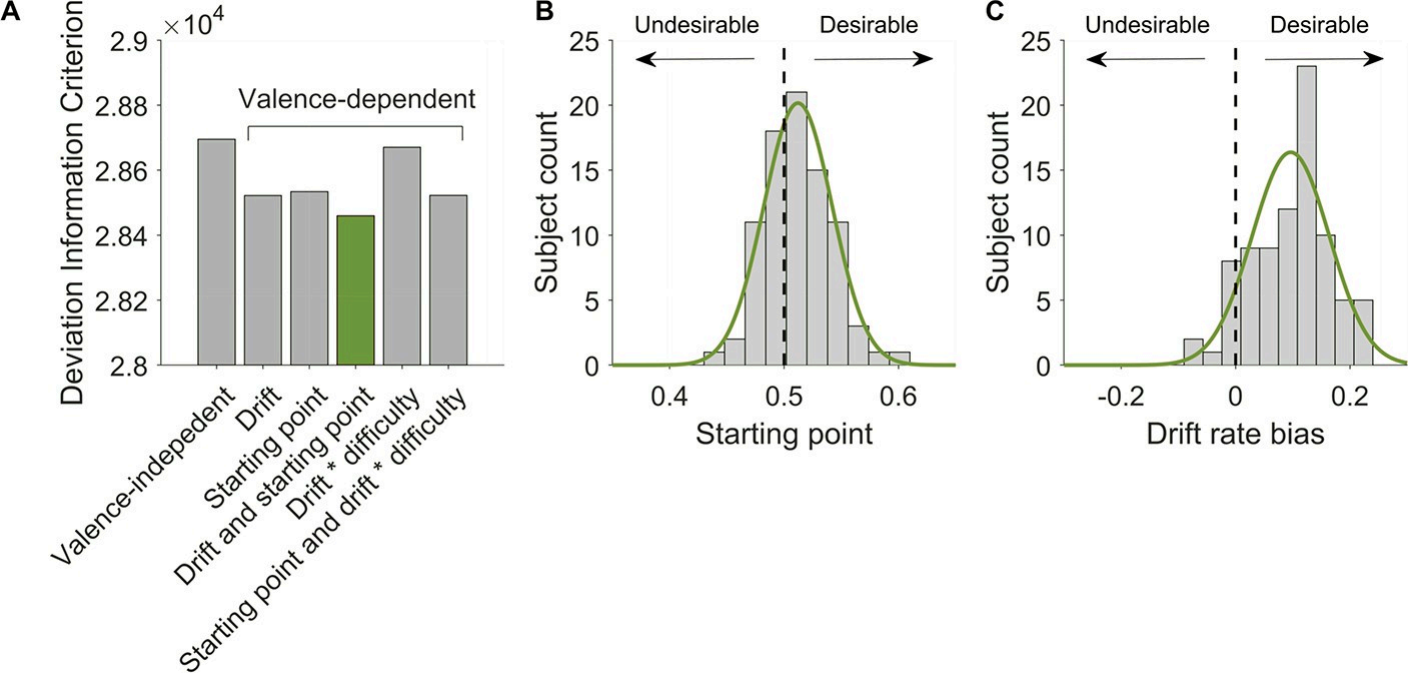
Drift-Diffusion model with valence-dependent starting point and drift rate provides the best fit. (**A**) Comparison of DIC scores reveals that all valence-dependent models perform better than the valence independent model. The same results were observed when comparing Bayesian Predictive Information Criterion scores [16], see Table 1. A model including a valence dependent drift rate and starting point outperformed all other tested specifications according to both measures. Models are ordered as in Table 1. (**B & C**) A histogram of individuals’ parameter estimates. The green line represents the best fitting normal distribution. The dashed line marks the value of unbiased parameters. (**B**) For 62% of participants, the estimated starting point was biased towards the desirable boundary (to the right of dashed line). (**C**) For 87% of participants, the estimated drift rate was greater when in the desirable than undesirable factory (bias to the right of dashed line).

The Deviance Information Criterion (DIC), a generalization of the Akaike Information Criterion for hierarchical models, was calculated for each model. The DIC scores indicated that Model 4, which included a valence dependent starting point and drift rate, outperformed all other models (**Fig 3A**). In this model the starting point (z) was significantly closer to the decision threshold for judging a factory as desirable (group level estimate *z* = 0.512, 95% CI [0.506, 0.519], significantly greater than a neutral starting point of 0.5). This pattern was observed in 62% of participants’ individual *z* estimates (**Fig 3B**). The bias in drift rate *β*_2_ was significantly greater than 0, such that drift rate was greater when in a desirable than undesirable factory (group level estimate *β*_2_ = 0.096, 95% CI [0.082, 0.111]). This pattern was observed in 87% of participants’ individual *β*_2_ estimates (**Fig 3C**). The bias in drift rate and starting point parameters were not significantly correlated (R = 0.15, p = 0.16). The results imply both that participants are poised to reach a desirable conclusion and that desirable evidence is given greater credence than undesirable evidence. These results suggest that evidence accumulation is valence dependent with motivation biasing both the starting point and drift rate. Using Bayesian Predictive Information Criterion (BPIC) for hierarchical models [16] instead of DIC revealed the same results (**Table 1**). BPIC applies a stronger penalty for model complexity.

**Table 1:** Drift diffusion model specifications.

Number	Model	Starting point (z)	Drift Rate (v)	DIC	BPIC
1.	**Valence independent**	*z* = 0.5	*v* = *β*_0_+*β*_1_*evidence*	28695	28937
2.	**Valence dependent** **drift rate**	*z* = 0.5	*v* = *β*_0_+*β*_1_*evidence*+*β*_2_*factory desirability*	28521	28821
3.	**Valence dependent starting point**	0<*z*<1	*v* = *β*_0_+*β*_1_*evidence*	28534	28828
4.	**Valence dependent drift rate and starting point**	0<*z*<1	*v* = *β*_0_+*β*_1_*evidence*+*β*_2_*factory desirability*	**28459**	**28790**
5.	**Valence dependent** **drift rate interacting with difficulty**	*z* = 0.5	*v* = *β*_0_+*β*_1_*evidence*+*β*_2_factory desirability * evidence	28670	28926
6.	**Valence dependent** **starting point and drift rate interacting with difficulty**	0<*z*<1	*v* = *β*_0_+*β*_1_*evidence*+*β*_2_factory desirability * evidence	28522	28831

Our replication study also returned an identical pattern of results—a DDM model in which drift rate and starting point were valence-dependent provided the best fit to the data (**supplementary material**).

To evaluate whether the above model specifications would benefit from including collapsing boundaries rather than a fixed decision threshold, we also fitted a model where the decision threshold was expressed as a Weibull cumulative distribution function (fit individually to each participant; see Methods). The results of this exercise suggest that the observed data was unlikely to be generated by a process with collapsing boundaries, as the model with fixed boundaries outperformed the model with collapsing boundaries both when participants judged a factory as desirable (AIC: fixed = -626.42, collapsing = -277.8616) and when judging a factory as undesirable (AIC: fixed = -597.38, collapsing = -263.2509). The parameters describing when the boundaries collapse (scale parameter difference between desirable and undesirable condition = 0.034, 95% CI [-0.66, 0.73], t(83) = 0.099, p = 0.92) and to what extent (scale parameter difference between desirable and undesirable condition = -0.66, 95% CI [-3.23, 2.02], t(83) = -0.49, p = 0.63) did not differ as a function of response type, suggesting that any observed biases were unlikely a result of a difference in collapsing decision thresholds.

#### Valence-dependent model provides better out of sample predictive accuracy than valence-independent model

**Fig 4:**
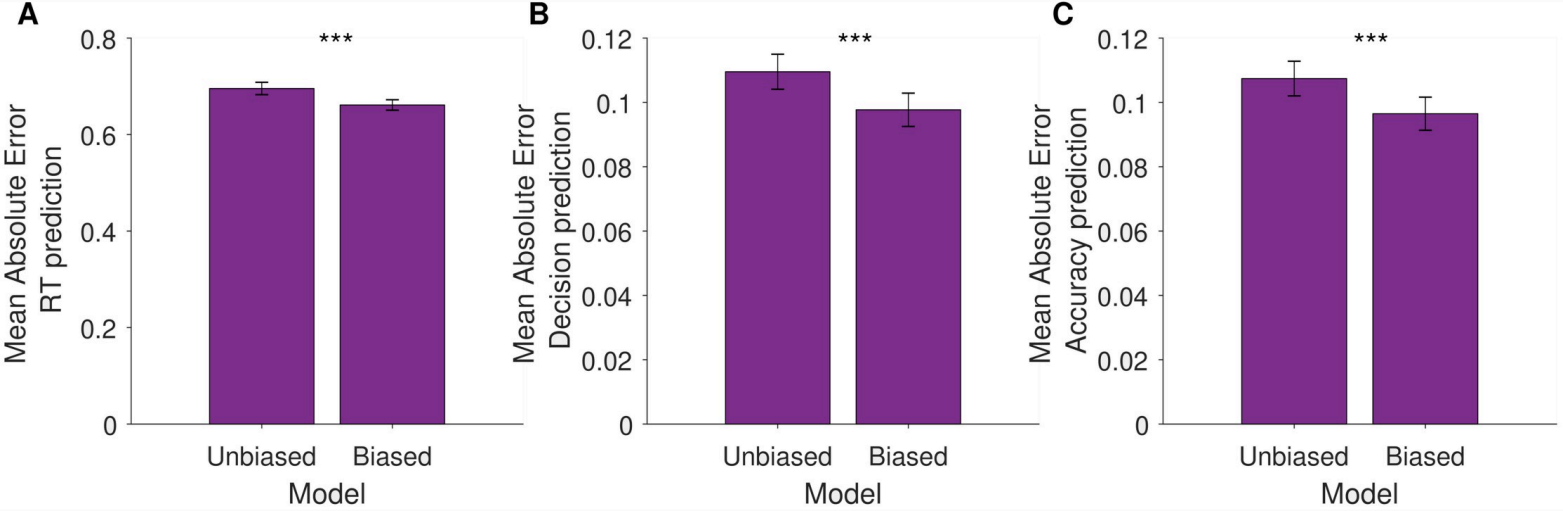
Valence-dependent model provides better predictive accuracy than valence-independent model. We simulated data on odd trials, based on parameter estimates obtained from fitting the data on even trials, separately for the winning valence-dependent model and the valence independent model. For each trial we calculated (**A**) the absolute difference between the observed RT and the simulated RT for each model and then averaged these quantities for each participant. We did the same for participants’ (**B**) judgments (i.e., desirable or undesirable responses coded as 1 and 0) and (**C**) accuracy (i.e., correct or incorrect responses coded as 1 or 0). For all three measures mean absolute errors were significantly lower for predictions arising from the valence-dependent model than the valence-independent model. *** P < 0.001, Error Bars SEM.

To test for predictive accuracy, we fitted both the winning model (which includes valence dependent drift rate and starting point) and the valence-independent model to data from even trials and evaluated how well the models predicted responses on odd trials using mean absolute error (MAE) as a measure of fit (**Fig 4**). The winning model predicted log reaction times better than the valence-independent model (MAE valence-dependent = 0.66, MAE valence-independent 0.70; comparison: t(3295) = -5.49, p < 0.0001), as well as judgements (MAE valence-dependent = 0.098, MAE valence-independent 0.110; comparison: t(3295) = -4.10, p < 0.0001) and accuracy (MAE valence-dependent = 0.097, MAE valence-independent = 0.108; comparison: t(3295) = -3.89, p < 0.0001). We fit a psychometric function to each of the model’s simulated responses. This clearly shows that while the valence dependent model reproduces the pattern of observed results (**Fig 1B;** indifference point for desirable β_0_ = 1.37, 95% CI [0.40, 2.33] vs. undesirable β_0_ = -1.91, 95% CI [-2.41, -1.42]), the valence independent model did not (**Fig 1C;** indifference point for desirable β_0_ = -0.17, 95% CI [-0.53 0.17] vs. undesirable β_0_ = -0.15, 95% CI [-0.47, 0.16])

## Discussion

The findings show that motivation has a profound effect on the process by which evidence is accumulated. On trials in which participants indicated they believed the state was desirable, they ceased gathering data earlier and required a smaller proportion of samples to be consistent with that conclusion. We used a computational model to characterize the underlying factors that may generate this behavior. The model revealed two factors; first, participants began the process of evidence accumulation with a biased starting point towards the desired belief. Thus, they required less evidence to reach that boundary. Second, the drift rate–the rate of information accumulation [14]–was greater on trials in which participants were in the desirable state than the undesirable state. If only a bias starting point was observed, this would have indicated that people might make fast errors, but with time/evidence would have corrected their initial biases. The existence of a process bias, however, makes correction more difficult. While participants incorporate both desirable and undesirable evidence into judgments, the larger weight assigned to desirable evidence means that biases could increase over time with more evidence accumulation. These results indicate that the temporal evolution of beliefs is influenced by what people wish to be true and that evidence accumulation is valence dependent. That is, the rules of accumulation depend on whether the data is favorable or unfavorable.

Most learning models [17–19] assume that agents learn from information they encounter, but that the learning process itself is not influenced by whether the evidence supports a desired or undesired conclusion. This study suggests this assumption is likely false. By allowing the parameters of a standard evidence accumulation model to vary as a function of the desirability of the evidence we were able to better explain and predict participants’ behavior. We chose to model the data with a drift-diffusion model because its components mapped onto the two alternatives of desirability bias in judgment. These components have been increasingly validated through targeted manipulations [20] and associated with specific neural and physiological correlates [21–25]. The good fit of the model to our data, as well as the alignment of the model results with the behavioral analyses vindicates the choice. We speculate that incorporating valence into other classes of learning models will also increase their predictive accuracy.

Our findings are in accord with previous suggestions that people hold positively biased priors [12] and update their beliefs more in response to good than bad news [13,26–29]. We speculate that biased evidence accumulation could be due to biases in perception [30, 31], attention [32, 33] and/or working memory [34, 35]. For example, participants may have attended to desirable stimulus to a greater extent than the undesirable stimulus, such that the former were assigned greater weight when forming beliefs. Such stimulus could also be maintained in working memory longer. These biases are thought to be automatic and do not require large cognitive resources [31, 36]. Here, we show such biases manifest into differential patterns of evidence sampling and accumulation. Our results also support a previous demonstration that people need less evidence to reach desirable conclusions in the domains of health and social interaction [9]. We go further in evidencing this in a situation where (i) participants are incentivized for accuracy, (ii) the desirable and undesirable conditions differ only on desirability and (iii) we provide insight to the underlying computations.

In sum, the current study describes how the motivation to hold a certain belief over another can decrease the need for supporting evidence. The implication is that people may be quick to respond to signs of prosperity (such as rising financial markets)–forming desirable beliefs even when evidence is relatively weak- but slow to respond to indictors of decline (such as political instability)–forming undesirable beliefs only when negative evidence can no longer be discarded. Indeed, in our study participants were more likely to hold positive false beliefs (falsely believing they are in the desirable factory when in fact they were in the undesirable factory) than negative false beliefs (falsely believing they are in the undesirable factory when in fact they were in the desirable factory). While both positive and negative false beliefs resulted in a material cost, we speculate that positive false beliefs may have non-monetary benefits. In particular, it has been hypothesized that beliefs, just like material goods and services, have utility in and of themselves [30–36]. In certain circumstances it is possible that the increase in utility from false beliefs themselves may be greater than the material utility lost, resulting in net benefit.

## Methods

### Participants

We recruited 100 participants (*M*_*age*_ = 34.48, 44% female) from Amazon Mechanical Turk (www.mturk.com). To qualify for participation, participants had to be resident in the United States. Participants were paid $4.5 for their participation and were promised an unspecified performance related bonus for a task that was expected to take 30 minutes. The study was approved by the ethics committee at University College London. Informed written consent was gained from participants.

### Procedure

#### Factory game task

Participants played 80 trials of the “Factory Game”. They began each trial by pressing the space bar, after which they witnessed an animated sequence of televisions and telephones passing along a conveyor belt. Each object would take 400 ms to traverse the belt with a 150 ms lag between stimuli.

There were two types of trials: Telephone Factory trials and Television Factory trials. In telephone factory trials the probability of each item in the animated sequence being a telephone was 0.6. and of being a television 0.4. For Television Factory trials the proportion was reversed. The current trial type was randomly determined with replacement on every trial with an equal probability for each trial type.

Participants were tasked with judging whether they were in a Telephone Factory trial or whether they were in a Television Factory trial. Since the trial type was not directly observable, their means of doing this was through reverse inference over the sequence of objects they were seeing. Participants were free to respond as soon as they wished after initiating the trial and the sequence would continue until they made their choice.

Participants began the game with an endowment of 5000 points. Each 100 points was worth 1 cent. One of the two factory types was randomly assigned per participant to be the desirable factory type and the other to be an undesirable type. Participants were informed that each time they visited the desirable factory, they would win an unspecified number of points, and each time they visited the undesirable factory, they would lose an unspecified number of points. *Crucially*, *this bonus was entirely outside of the participant’s control*, *i*.*e*. *it was not affected by the judgments the participant made*. Separately, participants were informed that they would earn an unspecified number of points for making a correct judgment and lose an unspecified number of points for making an incorrect judgment. The magnitude of each unspecified bonus/loss are independent of each other, potentially unequal and vary randomly on each trial.

We dropped trials where the participant made their judgment before seeing a second item. In cases where a participant did this in over half their trials, we assumed that participant was not appropriately engaging with the task and eliminated the entirety of their trials. We dropped 10 participants for this reason, as well as a further 123 responses made before seeing second item. We additionally excluded 3 participants whose average accuracy in the task was two standard deviations below the mean of the sample (i.e. for whom accuracy was below 53.28%; mean accuracy of the sample was 71.24%), assuming that these participants were guessing rather than providing their answers based on presented evidence. Finally, 3 participants were excluded as possible bots. These included "participants" who had at least two of the following indicators: nonsense answers to open-ended questions and/or IPs originating outside of the region targeted by Mturk and/or reaction times at regular intervals (i.e. button presses at exactly the same millisecond after the start of the trial) in more than 10% of trials and/or comprehension questions at chance level. After the above exclusions, we performed the analysis on 84 participants, and a total of 6597 trials. The same exclusion criteria are applied in the replication and control studies.

#### Training

Participants received extensive instructions prior to playing the game, and were required to answer multiple choice comprehension check questions on the key points of the task, with the question repeated until they either chose correctly or reached three times, upon which the correct answer was displayed to them. The comprehension check questions addressed the following key points of how the game worked: that telephone factories mostly produced telephones, but sometimes produced televisions; investment bonus was independent of the judgments they made; which factory was their desirable factory; and that trial types were randomly determined and it was not guaranteed that they would see exactly the same amount of each type of factory.

Participants then played a practice session of 20 trials, where the trial type was visibly displayed to them, so they could have prior experience of the outcome contingencies and the trial type distribution.

### Data analysis

#### Psychometric function

To relate participants’ judgments to the strength of evidence they observed we fitted a psychometric function, using a generalized mixed effects equivalent of a logistic regression, with fixed and random effects for all independent variables. We fitted these functions separately for participants for whom TV factory was desirable and for whom TV factory was undesirable.

$$P(TV)=\frac{1}{1+e^{-(\beta_{1}X-\beta_{0})}}$$

Where *P*(*TV*) is the probability of a participant indicating they are in a TV factory; *X* is the proportion of TV stimuli out of all stimuli observed on a trial. This variable was centred, thus ranging from 0.5 when all samples were TVs to -0.5 when all samples were phones; *β*_0_ is the indifference point–reflecting the proportion of TVs required to respond TV 50% of the time. If *β*_0_ = 0, participants would indicate they are in a TV factory half the time when half the samples were TVs. When *β*_0_ is low the function will move left and vice versa; *β*_1_ is the slope, reflecting by how much the probability of a participant indicating they are in a TV factory increases when the proportion of TVs increases by one unit.

#### RT and number of samples

As stimuli were presented at a steady pace, the number of samples drawn was highly correlated with reaction times (R = 0.99, p < 0.00001) and thus these two measures can be thought of as interchangeable. As the number of samples drawn before making a judgment was non-normally distributed and had a heavy positive skew, we log-transformed this variable [37].

#### Speed-accuracy trade-off

To examine speed-accuracy trade-off we divided the trials into fast and slow, based on median reaction time of the participant, and then calculated the average accuracy of desirable and undesirable responses within these categories. We performed a 2x2 ANOVA, with average accuracy as a dependent variable, and response (desirable/undesirable) and speed (fast/slow) as independent factors.

#### Drift-diffusion modelling

Our aim in modeling our task using the drift-diffusion framework was to assess the contribution of both the starting point and drift rate to the desirability bias we saw in our data. To that end, we implemented and compared six different specifications of a drift-diffusion model (DDM; see Table 2).

**Table 2:** Variants of drift-diffusion model.

Number	Model	Starting point (z)	Drift Rate (v)
1.	**Valence independent**	*z* = 0.5	*v* = *β*_0_+*β*_1_*evidence*
2.	**Valence dependent** **drift rate**	*z* = 0.5	*v* = *β*_0_+*β*_1_*evidence*+*β*_2_*factory desirability*
3.	**Valence dependent starting point**	0<*z*<1	*v* = *β*_0_+*β*_1_*evidence*
4.	**Valence dependent drift rate and starting point**	0<*z*<1	*v* = *β*_0_+*β*_1_*evidence*+*β*_2_*factory desirability*
5.	**Valence dependent** **drift rate interacting with difficulty**	*z* = 0.5	*v* = *β*_0_+*β*_1_*evidence*+*β*_3_factory desirability * evidence
6.	**Valence dependent** **starting point and drift rate interacting with difficulty**	0<*z*<1	*v* = *β*_0_+*β*_1_*evidence*+*β*_3_factory desirability * evidence

In particular, in models with valence-independent starting point its value was fixed at 0.5. In models with valence-dependent staring point, its value could vary between 0 and 1. In models with an unbiased drift rate the parameter was symmetric for desirable and undesirable factories (v and -v). In models with biased drift rate the model additionally included a term reflecting the difference between drift rates for desirable and undesirable factories (*β*_3_*factory desirability*). “Factory desirability”—is the true factory visited coded as 1 for desirable factories and 0 for undesirable factories. Moreover, following an approach used previously [18, 19], in all cases the drift rate was allowed to vary on each trial as a function of the proportion of samples observed that are consistent with the true state (*β*_1_evidence). This variable was centred, ranging from 0.5 when all samples were consistent with the true state to -0.5 when all samples were inconsistent with the true state. All models also included parameters for the decision threshold (α) and non-decision time (t0).

*β*_0_ is a constant.

*β*_1_ is the weight by which the evidence alters the drift rate.

*β*_2_ is a bias term reflecting an additional weight added to the drift rate as a function of the factory desirability. Positive values indicated a bias towards desirable judgements, and negative values indicated a bias towards undesirable judgements.

*β*_3_ is the weight put on the interaction term, allowing the evidence to alter the drift rate differently in desirable and undesirable factories.

We used the HDDM software toolbox [38] to estimate the parameters of our models. The HDDM package employs hierarchical Bayesian parameter estimation, using Markov chain Monte Carlo (MCMC) methods to sample the posterior probability density distributions for the estimated parameter values. We estimated both group-level parameters as well as parameters for each individual participant. Parameters for individual participants were assumed to be randomly drawn from a group-level distribution. Participants’ parameters both contributed to and were constrained by the estimates of group-level parameters.

In fitting the models, we used priors that assigned equal probability to all possible values of the parameters. Also, since our “error” RT distribution included relatively fast errors we included an inter-trial starting point parameter (*sz*) for both models to improve model fit [39]. We sampled 20000 times from the posteriors, discarding the first 5000 as burn in. MCMC are guaranteed to reliably approximate the target posterior density as the number of samples approaches infinity. To test if the MCMC converged within the allotted time, we used Gelman-Rubin statistic on 5 iterations of our sampling procedure. The Gelman–Rubin diagnostic evaluates MCMC convergence by analyzing the difference between multiple Markov chains. The convergence is assessed by comparing the estimated between-chains and within-chain variances for each model parameter. In each case, the Gelman-Rubin statistic was close to one (<1.1), suggesting that MCMC were able to converge. To assess if the parameters describing the bias in prior and drift rate are significantly different from a valence-independent specification of the model, we compared 95% confidence intervals of the parameters’ values against the theoretically unbiased values.

In addition, model fits were compared using the Deviance information criterion, which is a generalization of the Akaike Information Criterion (AIC) for hierarchical models. The DIC is commonly used when the posterior distributions of the models have been obtained by Markov chain Monte Carlo (MCMC) simulation. It allows one to assess the goodness of fit, while penalizing for model complexity [40].

#### Cross-validation

To further validate the model and check its predictive accuracy, we fitted again the valence dependent and valence independent models using data from only even trials. We then used the parameter estimates to predict log RTs, judgments and their accuracy for odd trials for each participant. The simulation was repeated 1000 times with normally distributed random noise added to the drift rate averaging predicted responses for each trial. We then calculated mean absolute error between predicted and observed responses (RTs, judgments and judgment accuracy). We compared the average mean absolute errors between the models using a paired t-test. We also fitted a psychometric function to the simulated data.

#### Collapsing boundaries

Decision boundaries may collapse over time rather than remain fixed, reflecting increasing impatience or urgency of decisions [41, 42]. To investigate if such a model fits our data we fitted a pure diffusion model with a fixed decision threshold and a diffusion model with a collapsing boundary, modeled as a Weibull cumulative distribution function [41]:

$$u_{t}=a-[1-\exp\left(-{\left(\frac{t}{\lambda }\right)}^{k}\right)]∙(a-a^{\prime })$$

Where *u*_*t*_ is a threshold at time *t*, *a* is the initial value of the boundary, *a'* is the asymptotic value of the boundary (i.e. the extent to which the boundary collapses), *λ* and *k* are the scale and shape parameters of the Weibull function, influencing the stage at which the boundary starts to collapse and the steepness of the collapse, respectively. The shape parameter *k* was fixed to 3, corresponding to a “late collapse” decision strategy, following other studies showing that it’s a typical strategy implemented by participants [41].

A judgment is made when the accumulated difference between the number of samples supporting one type of the factory over the other exceeded one of two symmetric boundaries, *±u*_*t*_. The accumulated difference was computed as:

$$X_{t}=X_{t-1}+d_{t}+\varepsilon_{t},\varepsilon ∼N(0,\sigma^{2})$$

Where *d*_*t*_ is the difference between number of evidence points at time *t*, and *ε*_*t*_ is a random noise sampled from a normal distribution with a mean of 0 and variance of *σ*^2^. *X*_1_ denoted a bias in a starting point.

Model parameters were fitted to each participant’s data for desirable and undesirable responses separately using maximum likelihood estimation method. For each trial, we simulated the models 1000 times for a given set of proposal parameters and calculated the proportion of trials in which the model RT matched the empirical data. Denoting this proportion by *p*_*i*_, we maximized the likelihood function *L*(*D*|*θ*) of the data (*D*) given a set of proposal parameters (*θ*), by:

$$L(D|\theta )=\prod_{i=1}^{N}p_{i}$$

To find the best set of proposal parameters we first used an adaptive grid search algorithm and then used the five best sets of proposal parameters as starting points to a Simplex minimization routine [43]. In order to evaluate the quantitative fits of the models, we used Akaike Information Criterion.

## Supporting information

S1 Text Replication and extension experiment. (DOCX)

S2 Text Control experiment. (DOCX)
