# Supplementary material for: Evidence accumulation is biased by motivation: A computational account
Source: PLoS Comput Biol. 2019 Jun 27;15(6):e1007089. doi: 10.1371/journal.pcbi.1007089 (PMC6597032; doi:10.1371/journal.pcbi.1007089)
Supplement: S1 Text — (DOCX) [file pcbi.1007089.s001.docx]

**S1 Text - Extension & Replication experiment**. The findings of the main experiment were replicated using the same exact procedure except for two changes to the incentive structure:

1. Participants were informed the exact number of points they would win when visiting a desirable factory and lose when visiting an undesirable factory (100 points). Each 100 points was worth 1 cent.
2. Participants were informed they would receive 30 points for each accurate judgement. There was no penalty for inaccurate judgments.

We tested 100 participants using the same exclusion criteria as for the main experiment, that resulted in a final sample of 92 participants (7 participants were excluded due to more than half of the responses made before seeing the second stimuli and 1 person was excluded due to an average accuracy below two standard deviations from the mean accuracy observed in the sample).

The proportion of factories participants judged as desirable was significantly different from the proportion they actually encountered (*t*(91) = 3.85, p < 0.0001). They gathered less samples before concluding they were in a desirable than undesirable factory (*t*(91) = -4.01, p < 0.0001) and require a lower proportion of samples to be consistent with their judgment when reaching the desirable than undesirable conclusion (desirable: β_0_ = 0.37, 95% CI [0.16, 0.58], undesirable: β_0_ = -0.37, 95% CI [-0.56 -0.17]). They were more likely to falsely believe they were in a desirable factory when in fact they were in an undesirable factory, than to falsely believe they were in an undesirable factory when in fact they were in a desirable factory (*t*(91) = 4.41, *p* < 0.0001).

The valence-dependent drift diffusion model including biased starting point and biased drift, provided a better fit than the valence independent model (DIC _valance-dependent_  = 34898.05, DIC _valance-independent_  = 35162.67). In the valence-dependent DDM model, the starting point (z) was significantly closer to the decision threshold for judging a factory as desirable (group-level estimate: 0.520, 95% CI [0.513, 528]). The estimated drift rate bias was significantly greater than 0, such that drift rate was greater when in a desirable than undesirable factory (group-level estimate: 0.057, 95% CI [0.047, 0.067]). 73% of participants had a starting point above the neutral point of 0.5, and 89% of participants had a drift bias above the neutral point of 0.
