# Supplementary material for: Evidence accumulation is biased by motivation: A computational account
Source: PLoS Comput Biol. 2019 Jun 27;15(6):e1007089. doi: 10.1371/journal.pcbi.1007089 (PMC6597032; doi:10.1371/journal.pcbi.1007089)
Supplement: S2 Text — (DOCX) [file pcbi.1007089.s002.docx]

**S2 Text - Control experiment.** The goal of the control experiment was to test whether the desirability bias we observed could be explained in terms of risk seeking. In particular when a participant judges a factory to be desirable they may either be correct in which case they will receive X+W points, or incorrect, in which case they will lose -Y-Z points. When a participant judges a factory to be undesirable they may either be correct in which case they will receive -Y+W points, or incorrect, in which case they will receive X-Z points. Thus responding “desirable” is associated with a larger range of outcomes than responding “undesirable”. To examine if this may be underlying the results we re-run the study with one change:

1. We reworded the instructions to explicitly emphasize that one response was associated with a higher spread of possible outcomes than the other response. We did not explicitly emphasize that being in one state is more desirable than the other. Note, that the incentive structure was the same and all the information was given to the participants.

We tested 100 participants on “The Factory Game”, using the same exclusion criteria as for the main experiment, that resulted in a final sample of 90 participants (5 participants were excluded due to more than half of the responses made before seeing second stimuli, another 5 were excluded due to average accuracy below two standard deviations from the mean accuracy observed in the sample).

The proportion of factories participants judged as desirable was not significantly different than the proportion they actually encountered (*t*(89) = 0.54, p = 0.59). They did not gather a different number of samples before concluding they were in a desirable than undesirable factory (*t*(89) = -0.97, p = 0.34) and did not require a different proportion of samples to be consistent with their judgment when reaching the two conclusions (desirable: β_0_ = -0.03, 95% CI [-0.25, 0.19] undesirable: β_0_ = -0.13, 95% CI [-0.32, 0.05] ). They were not more likely to falsely believe they were in a desirable factory when in fact they were in undesirable factory than to falsely believe they were in an undesirable factory when in fact they were in a desirable factory (*t*(89) = 0.72, *p* = 0.47). We thus conclude that differential spread of outcomes does not produce a bias in judgment of evidence accumulation in this task.
